# Necrotizing soft-tissue infections in pediatric intensive care: a prospective multicenter case-series study

**DOI:** 10.1186/s13054-021-03562-0

**Published:** 2021-04-12

**Authors:** Stéphane Dauger, Renaud Blondé, Olivier Brissaud, Marie-Odile Marcoux, François Angoulvant, Michael Levy, Groupe Francophone de Réanimation et Urgences Pédiatriques (GFRUP), Laurent Balu, Hélène Gatti, Etienne Javouhey, Nicolas Joram, Fabrice Lesage, Christophe Milesi, Marie-Hélène Perez, Isabelle Wroblewski, Réseau Mères-Enfants de la Francophonie (RMEF), Marc-André Dugas, Etienne Javouhey, Nicolas Joram, Fabrice Lesage

**Affiliations:** 1 Pediatric Intensive Care Unit, Assistance Publique-Hôpitaux de Paris, Service de Médecine Intensive - Réanimation Pédiatriques, Hôpital Universitaire Robert Debré, Université de Paris, 48 boulevard Sérurier, 75019 Paris, France; 2 Université de Paris, NeuroDiderot, INSERM UMR 1141, 75019 Paris, France; 3 Adult and Pediatric Intensive Care Unit, Centre Hospitalier de Mayotte, 97600 Mamoudzou, France; 4 Pediatric Intensive Care Unit, Hôpital Universitaire Pellegrin, Université de Bordeaux, 33000 Bordeaux, France; 5 Pediatric Intensive Care Unit, Hôpital Universitaire Purpan, Université de Toulouse, 31000 Toulouse, France; 6 Pediatric Emergency Department, Assistance Publique-Hôpitaux de Paris, Hôpital Universitaire Necker-Enfants Malades, Université de Paris, 75005 Paris, France; 7 Institut Pasteur, Invasive Bacterial Infection Unit, 75724 Paris, France

To the Editor,

Necrotizing soft-tissue infections (NSTIs) are severe diseases with documented high morbidity and mortality rates in adults [1]. In pediatric patients, data are scant and prospective studies extremely scarce [2]. Eneli et al. [3] reported 36 cases collected prospectively in Ontario from 2001 to 2003. The largest studies used North-American databases and exhibited the biases inherent in this methodology [4, 5]. We designed a prospective international observational study of all severe NSTI cases seen in pediatric intensive care units (PICUs), to evaluate the outcomes and treatments.

From February 2011 to July 2016, we included consecutive patients with NSTI aged 1 month to 18 years and admitted to 33 PICUs located in high-resource countries (continental France and French overseas territories, Switzerland, Canada, and The Netherlands). Institutional review board approval (IRB-0006477) and parental consent to data recording were obtained. NSTIs are infections of any of the soft tissue layers during which tissue necrosis occurs. For this study, we defined NSTI as painful, rapidly progressive, superficial, spreading erythema or skin necrosis, with laboratory evidence of inflammation and a fever (> 38.5 °C) or hypothermia (< 36 °C). We collected demographics; clinical, laboratory, and bacteriological data; radiological findings; and medical and surgical treatments. Descriptive data, collected without a statistical analysis plan, are reported as median [25th; 75th centiles], mean ± SD, or *n* (%).

In the 50 patients included during the 4.5-year period (Table 1), time from symptom onset to PICU admission was 2 [1; 2] days. Triggers were postoperative care (*n* = 12; 24%) and trauma or animal bite (*n* = 11; 22%). Severe comorbidities included cancer (*n* = 10; 20%), varicella (*n* = 9; 18%), and preexisting chronic skin disease (*n* = 3; 6%). No predisposing factors were identified in 8 (16%) patients. At diagnosis, 10 (20%) patients had received non-steroidal antiinflammatory drugs, 5 (10%) immunosuppressive agents, and 3 (6%) glucocorticoids. Most patients had severe critical illness at admission, often with respiratory and circulatory failure, translating into high mortality prediction scores. Bacteria were recovered from 43 (86%) patients in blood cultures (*n* = 16; 32%), skin swabs (*n* = 16; 32%), and skin biopsies (*n* = 4; 8%) (Table 2). Treatment combined respiratory support (58%), hemodynamic support (70%), antitoxin antibiotic (84%), and surgery (68%) (Table 2). Three (6%) patients died; all had severe comorbidities (mitochondrial cytopathy, solid tumor, and leukemia). Most patients had long hospital stays. Our sample was too small to identify factors associated with surgery or mortality.

**Table 1:** Characteristics of the study patients during the first 24 h after admission

Parameters	Values
Age (months)	60 (17.8; 128.5)
Weight (kg)	17.5 (11; 42)
Males/females	21 (42%)/29 (58%)
In-hospital patients	25 (50%)
Scores on admission	
PRISM-3 (first day in PICU)	9.5 ± 15.9
PIM-2 (first hour in PICU)	12.6 ± 23.3
PELOD (first day in PICU)	11.6 ± 25.1
POPC	1.6 ± 1.1
Clinical presentation	
Body temperature (°C)	38.2 (37; 38.8)
Respiratory distress	33 (66%)
Hypotension for age	28 (56%)
Oliguria (< 1 mL/kg/h)	14 (28%)
Main primary skin lesion	
Erythrosis	27 (54%)
Necrosis	13 (26%)
Bullae	10 (20%)
Surface (% of total body surface)^a^	6 (3; 10)
Location	
Legs	13 (26%)
Arms	12 (24%)
Head/neck	12 (24%)
Chest/abdomen	13 (26%)
Laboratory tests	
White blood cells (/mm^3^)	9 610 (5 445; 18 820)
Hemoglobin (g/dL)	9.7 (8.9; 11.2)
Platelets (/mm^3^)	187 500 (70 000; 305 250)
CRP (mg/L)	176 (85; 289)
Fibrinogen (g/L)	5 (3.7; 6.5)
Lactatemia (mmol/L)	2 (1.4; 3.8)
Total proteins (g/L)	52.5 (43.8; 63)
Urea (mmol/L)	4 (2.6; 5.9)
Creatinine (μmol/L)	28 (21; 57.5)

PRISM-3: Paediatric Risk of Mortality measured during the first 24 h after PICU admission; PIM-2: Paediatric Index of Mortality measured during the first hour after PICU admission; PELOD: Paediatric Logistic Organ Dysfunction, measured during the first 24 h after PICU admission; POPC: Paediatric Overall Performance Category

A few data were missing: CRP: 3, Fibrinogen: 7, Lactatemia: 1, Total proteins: 2, Urea: 1, and Creatinine: 1

^a^Lund and Browder chart. Data are reported as median [25th; 75th centiles], mean ± SD, or *n* (%)

**Table 2:** Assessments, treatments, and course of the disease during the PICU stay

Parameters	Values
Radiological assesment	
Ultrasound	21 (42%)
CT scan	22 (44%)
MRI	7 (14%)
Identified bacteria	43 (86%)
Only one micro-organism/two or more micro-organisms	36 (72%)/7 (14%)
Gram positive	40 (91%)
*Staphylococcus aureus*	17 (39%)
Methicillin-sensitive	15 (88%)
Panton valentine Leukocidin	15 (88%)
Toxic shock syndrome toxin	10 (59%)
Group A β-hemolytic *Streptococcus*	14 (32%)
Gram negative	12 (25%)
*Escherichia coli*	5 (11%)
*Pseudomonas aeruginosa*	4 (9%)
General treatments in the PICU	
Mechanical ventilation/duration (days)	29 (58%)/3 (2; 3)
Fluid expansion/volume (mL/kg)	35 (70%)/37.5 (22.8; 65)
Vasopressors/duration (days)	27 (54%)/3 (2; 3)
Morphine/duration (days)	43 (86%)/5 (4; 10.5)
Benzodiazepine/duration (days)	33 (66%)/5.5 (2; 9)
Transfusion of blood products	25 (50%)
Transfusion of albumin	25 (50%)
Specific treatments	
Surgery/time since admission (days)	34 (68%)/1 (1; 5)
One surgical procedure	15 (44%)
Multiple surgical procedures/median per patient	19 (56%)/3.5 (2; 5)
Amputation	0
Antitoxin antibiotics	42 (84%)
Immunoglobulins/doses (g/kg)^a^	18 (36%)/2 (2; 2)
Hyperbaric oxygen	5 (10%)
On PICU discharge	
Deep thrombosis	6 (12%)
Nosocomial infections	12 (24%) during 4 stays
In-hospital death/day of death	3 (6%)—D7, D9, and D13
POPC (without the 3 deaths)	1.7 ± 0.9
In-PICU length of stay (days)	12.5 ± 14.5; 8 (5; 14)
In-hospital length of stay (days)	35.6 ± 42; 21 (12.5; 41.8)

POPC: Pediatric Overall Performance Category; PICU: pediatric intensive care unit; Data are reported as median [25th; 75th centiles], mean ± SD, or *n* (%)

^a^Immunoglobulins (1 g/kg twice in 15 patients and 1 g/kg once in 3 patients) were started before surgery in patients with suspected toxic shock

The results of this largest prospective study of pediatric NSTIs to date confirm that this disease is rare in PICUs [2–6]. Predisposing conditions have shifted from varicella to healthcare and trauma [2, 3]. The percentage of body surface area involved on PICU admission was consistent with studies in adults [1], whereas a major difference in absolute value was noted (about 5 cm^2^ in children vs*.* 12 cm^2^ in adults), which may contribute to diagnostic delays in children. MRI was rarely used to assess lesion depth. As reported by others [2, 6], surgery was performed in two-thirds of patients, usually within 24 h, and aggressive medical treatments were used. This may explain the low mortality rate, in agreement with recent data [6], despite the high predicted mortality on admission. Most NSTIs were monobacterial and many were due to Gram-positive organisms, including methicillin-resistant *S. aureus* in a few patients, as previously reported [3]. Polyvalent immunoglobulin therapy was often used, perhaps due to frequent circulatory failure suggesting toxic shock, and might also have influenced mortality rates, as suggested in a recent adult study [1]. Strong collaboration between surgeons, anesthesiologists, and pediatric intensivists should be the cornerstone of NSTI management.

## Data Availability

The datasets used and/or analysed during the current study are available from the corresponding author on reasonnable request.

## Abbreviations

NSTI: Necrotizing soft-tissue infections
PICU: Pediatric intensive care unit
PRISM-3: Paediatric risk of mortality, measured during the first 24 h after PICU admission
PIM-2: Paediatric index of mortality, measured during the first hour after PICU admission
PELOD: Paediatric logistic organ dysfunction, measured during the first 24 h after PICU admission
POPC: Pediatric overall performance category

## References

[1] Madsen MB, Skrede S, Perner A (2019). Patient's characteristics and outcomes in necrotising soft-tissue infections: results from a Scandinavian, multicentre, prospective cohort study. Intensive Care Med.

[2] Schröder A, Gerin A, Firth GB, Hoffmann KS, Grieve A, Oetzmann von Sochaczewski C (2019). A systematic review of necrotising fasciitis in children from its first description in 1930 to 2018. BMC Infect Dis.

[3] Eneli I, Davies HD (2007). Epidemiology and outcome of necrotizing fasciitis in children: an active surveillance study of the Canadian Paediatric Surveillance Program. J Pediatr.

[4] Totapally BR (2017). Epidemiology and outcomes of hospitalized children with necrotizing soft-tissue infections. Pediatr Infect Dis J.

[5] Endorf FW, Garrison MM, Klein MB, Richardson A, Rivara FP (2012). Characteristics, therapies, and outcome of children with necrotizing soft tissue infections. Pediatr Infect Dis J.

[6] Zundel S, Lemaréchal A, Kaiser P, Szavay P (2017). Diagnosis and treatment of pediatric necrotizing fasciitis: a systematic review of the literature. Eur J Pediatr Surg.

